# Does microfluidic sperm selection improve clinical pregnancy and miscarriage outcomes in assisted reproductive treatments? A systematic review and meta-analysis

**DOI:** 10.1371/journal.pone.0292891

**Published:** 2023-11-20

**Authors:** Janaina Ferreira Aderaldo, Karina da Silva Maranhão, Daniel Carlos Ferreira Lanza

**Affiliations:** 1 Januário Cicco Maternity School of Brazilian Company of Hospital Services (MEJC/UFRN-Ebserh), Natal, Brazil; 2 Biochemistry Department, Federal University of Rio Grande do Norte–UFRN, Natal, Brazil; 3 Independent Researcher, Brazil; University of Port Harcourt, NIGERIA

## Abstract

**Background:**

The microfluidic sperm selection (MFSS) device has emerged as a promising adjunct in assisted reproduction treatments (ART). It employs mechanisms of biomimicry based on the microanatomy of the female reproductive tract through strategies like chemotaxis and rheotaxis. Numerous studies assert improvements in ART outcomes with the use of MFSS, often attributed to the theoretical reduction in sperm DNA damage compared to other techniques. However, these attributed benefits lack validation through large-scale clinical trials, and there is no significant evidence of enhanced assisted reproductive treatments (ART) outcomes.

**Objective:**

To evaluate whether the utilization of MFSS enhances clinical pregnancy results and abortion outcomes in couples undergoing ART compared to standard sperm selection techniques for Intracytoplasmic Sperm Injection (ICSI). We also assessed laboratory outcomes as a supplementary analysis.

**Search methods:**

We conducted searches across databases including PubMed, NIH, LILACS, CENTRAL, Crossref, Scopus, and OpenAlex. A total of 1,255 records were identified. From these, 284 duplicate records were eliminated, and an additional 895 records were excluded due to their association with patent applications, diagnostic tests, forensic analyses, or irrelevance to the research focus. Among the initially eligible 76 studies, 63 were excluded, encompassing abstracts, studies lacking adequate control groups, and ongoing clinical trials. Ultimately, 13 studies were selected for inclusion in the ensuing meta-analysis.

**Results:**

Regarding clinical pregnancy, we assessed a total of 868 instances of clinical pregnancies out of 1,646 embryo transfers. Regarding miscarriage, we examined 95 cases of pregnancy loss among the 598 confirmed clinical pregnancies in these studies.

**Conclusion:**

The utilization of MFSS demonstrates marginal positive outcomes compared to standard sperm selection techniques, without statistical significance in any of the analyses.

**Broader implications:**

This study conducted the first meta-analysis to evaluate clinical pregnancy rates, miscarriage rates, and laboratory results associated with the use of MFSS compared to standard sperm selection techniques. We have also listed potentially eligible studies for future inclusion. It’s important to emphasize the need for multicenter studies with standardized parameters to attain a more robust clarification of this issue.

## Introduction

Infertility is defined as the inability to achieve a pregnancy after 1 year of regular attempts [1], or due to physio-psychological conditions that render intercourse or the matching of gametes incompatible [2]. Presently, approximately 15–20% of couples worldwide face infertility [3, 4]. This percentage is on the rise [1, 5], attributed to various factors including lifestyle choices, prevalent diseases, and the trend of delaying parenthood [6, 7]. It’s estimated that male-related issues contribute to about 50% of couple infertility cases, with 20–30% of these attributed to associated factors [8]. Yet, even with significant advancements and the introduction of numerous add-ons over the past two decades, live birth rates have only stabilized at around 33% per cycle [9, 10], translating to about 5% per retrieved oocyte [11, 12], marking the process as relatively inefficient.

Though there isn’t a universally accepted definition for "add-ons", they’re generally understood to be supplementary tools or methods like equipment, lab procedures, surgical processes, and additional clinical elements not essential to standard protocols [7, 13, 14]. The adoption of these add-ons in Assisted Reproductive Treatments (ART) has surged, prompting the Cochrane Gynaecology and Fertility Group to release a special compilation in October 2020, detailing the efficacy of these innovations [15].

When it comes to sperm selection for Intracytoplasmic Sperm Injection (ICSI), the commonly accepted methods are Density Gradient Centrifugation (DGC) and swim-up [16]. A notable innovation in this area is the microfluidic sperm sorter (MFSS) [16, 17], designed to emulate the female reproductive tract’s microanatomy using strategies like chemotaxis and rheotaxis [16].

While microfluidics is viewed as an established technology in various biomedical fields [10], it’s still regarded as a promising emerging technique for sperm selection, primarily due to its adaptability, automation, precision, and speed [12]. Notwithstanding its theoretical advantages, notably in reducing sperm DNA damage [12, 18], there’s still a conspicuous lack of compelling evidence suggesting improved clinical pregnancy rates without associated miscarriage when using MFSS [7, 10].

To conclude, this study aims to assess the clinical outcomes, specifically pregnancy and miscarriage rates, in ART treatments using MFSS for sperm selection, juxtaposed against conventional techniques like DGC and swim-up. The study’s relevance is amplified by the rising adoption of this technology, despite the absence of robust evidence supporting its clinical efficacy.

## Methods

### Search strategy and eligibility criteria

This research adhered to the PRISMA guidelines [19] (refer to S1 Table) and was registered with PROSPERO under the identifier CRD42022314968. Our search strategy was framed using the PICOT [20] acronym to provide clear direction:

- (P) Population: Infertile couples submitted to ART treatment with ICSI;
- (I) Intervention: Sperm selection by MFSS;
- (C) Comparison: Density Gradient Centrifugation (DGC); swim-up; Magnetic Activated Cell Sorting (MACS); Microdissection testicular sperm extraction (micro-TESE); testicular sperm aspiration (TESA);
- (O) Outcomes: clinical pregnancy; miscarriage; fertilization, cleavage, blastocysts rates; and
- (T) Time: none.

We conducted a systematic search in Pubmed, National Institutes of Health (NIH) ‐ Clinical Trials, Scientific health information from Latin America and the Caribbean countries (LILACS), Cochrane Central Register of Controlled Trials (CENTRAL), Embase (n = 395), and Google Scholar.

The inclusion criteria for the study selection comprised: a) primary and peer-reviewed scholarly articles; b) comparative analyses involving MFSS and alternative sperm selection methodologies; c) utilization of intracytoplasmic sperm injection (ICSI) for insemination; and d) experimentation conducted on human subjects.

The exclusion criteria were as follows: a) diagnostic examinations; b) patent applications; c) investigations focusing on obstructive azoospermia; d) studies with the primary objective of assessing sperm quality parameters such as morphology, motility, and DNA fragmentation.

### Selection process

The results were compiled in the Endnote X9 reference manager [21], simplifying the process of removing duplicates. Two independent reviewers, K.S.M. and J.F.A., assessed the studies based on their titles and abstracts using the Rayyan tool [22], strictly following the set inclusion and exclusion criteria. All discrepancies between the reviewers were addressed through mutual discussion.

### Data extraction

Selected articles deemed appropriate for in-depth analysis underwent manual and independent data extraction. The data extraction template encompassed the following specifics: authors, year of publication, location of the study, research design, number of participants, type of MFSS used, comparison method, number of successful pregnancies, and occurrences of miscarriages. Studies that advanced to the previous stage but lacked clear or extractable raw data, and which didn’t respond to efforts for additional information, were consequently excluded.

### Quality assessment and risk of bias

The risk of bias analysis was carried out by two separate reviewers (K.S.M.; J.F.A.), using the Newcastle-Ottawa approach [23]. Each study’s quality assessment was categorized across three dimensions: selection, comparability, and outcome, in accordance with the classification provided by the Agency for Healthcare Research and Quality (AHRQ) methodology [24].

### Statistics for meta-analysis

The compiled data was structured and presented as dichotomous variables. The statistical framework for the meta-analysis was executed using RevMan version 5.4 [25]. To accommodate potential disparities among the studies, we gauged heterogeneity with the inconsistency index (I²) and adopted a random-effects model complemented by subgroup analysis. We chose the risk ratio (RR) as the metric, using the Mantel-Haenszel approach [26].

Taking into consideration the potential impact of study sample sizes, our statistical model factored in this influence, represented by the breadth of the 95% confidence interval (CI). Additionally, we performed sensitivity tests to identify specific studies that might have a pronounced bearing on the overarching findings.2

For clinical outcomes, our measures included:

1. Clinical pregnancy rate, deduced as the fraction of validated gestational sacs or detectable heartbeats via ultrasound against the entirety of embryo transfers [2].
2. Miscarriage rate, gauged by tallying miscarriage occurrences against the full count of clinical pregnancies.

For laboratory outcomes, we referenced these criteria:

1. Fertilization rate, pinpointed from the ratio of efficaciously fertilized oocytes (attaining the 2PN phase) against all inseminated oocytes.
2. Cleavage rate, ascertained by dividing the number of split embryos by all inseminated oocytes.
3. Blastocyst rate, reflecting the percentage of blastocysts harvested from all inseminated oocytes.

### Reliability of the evidence

To evaluate the caliber of the evidence uncovered, we utilized the Grading of Recommendations, Assessment, Development, and Evaluations (GRADE) framework [27, 28]. The GRADE methodology was devised to facilitate the assessment and presentation of evidence quality and to guide the formulation of recommendations in clinical guidelines, systematic reviews, and meta-analyses [27].

## Results

### Study selection

We executed the search strategies (S2 Table) across six predefined databases, yielding a total of 11,593 records: Pubmed (n = 9018); NIH (n = 7); LILACS (n = 16); CENTRAL (n = 160); Embase (n = 395); and Google Scholar (n = 1997).

From this pool, 1284 records were identified as duplicates and subsequently eliminated. An additional 1233 entries were dismissed because they pertained to patent filings, diagnostic procedures, forensic evaluations, or they didn’t align with the study’s objective. Furthermore, research utilizing microfluidics to evaluate sperm DNA integrity or seminal attributes were also set aside.

**Fig 1:**
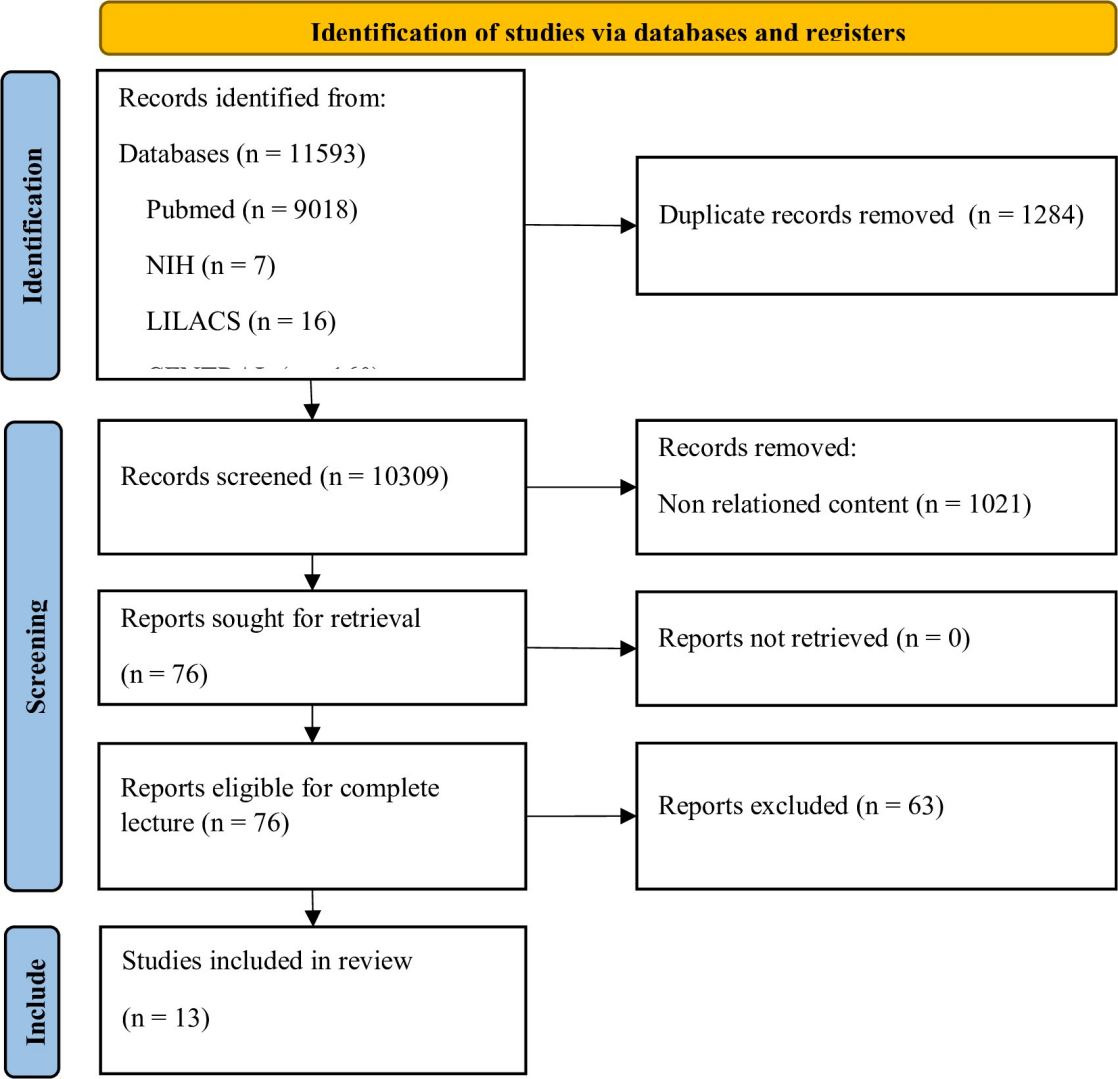
PRISMA diagram of records selection. We identified 11,593 records across 6 databases, out of which 1284 duplicate records were removed. We excluded 1233 records that were related to patent applications, diagnostic tests, forensic analysis, and irrelevant content. Among the 76 potentially eligible studies, 63 were excluded due to being abstracts, lacking an adequate control group, or being ongoing clinical trials. Ultimately, we included 13 studies for this meta-analysis.

From the initially identified 76 studies, 63 were ruled out, including mere abstracts, research lacking adequate control groups, and currently active clinical trials. This left 13 studies deemed appropriate for inclusion in the ensuing meta-analysis (Fig 1 and Tables 1 and S3).

**Table 1:** Eligible studies about MFSS in ART outcomes.

Study identification			Study design										Outcomes		
Author	Year	Country	Time	Infertility factor	Randomization	Previous ART	same couples for intervention and control	successive stimulus in the same group	Sibling oocytes	Insemination method	Type of MFSS	Control type	Laboratory	Clinical	Embryo biopsy
Kalyan *et al*.	2019	Turkey	Prospective	severe mal infertility was excluded	Y	*	Y	N	Y		Fertile Plus®	swim-up	Y	Y	Y
Yetkinel *et al*.	2019	Turkey	Prospective	unexplained	Y	*	N	N	N	ICSI	Fertile Chip®	swim-up	Y	Y	N
Yildiz *et al*.	2019	Turkey	Prospective	Unexplained; recurrent failure	Y	N / Y	N	N	N	ICSI	Fertile Plus®	DGC	Y	Y	N
Tavares *et al*.	2020	USA	Prospective	recurrent failure; high sperm DNA fragmentation	*	Y	N	N	N	ICSI	*	micro-TESE	Y	Y	**
Anbari *et al*.	2021	Iran		Unexplained; recurrent failure; low number of oocytes	Y	Y	N	N	N	ICSI + MSOME	*	swim-up	Y	Y	N
Guler *et al*.	2021	Turkey	Prospective	male with astheno-teratozoospermia	Y	*	Y	N	Y	ICSI	Fertile Ultimate®	DGC	Y	N	N
Leisinger *et al*.	2021	USA	Prospective	severe male factor excluded	Y	*	Y	N	Y	ICSI	ZyMōt™	DGC / swim-up	Y	Y	Y
Ozcan *et al*.	2021	Turkey	Retrospective	male infertility factor	N	Y	N	N	N	ICSI	Fertile Plus®	DGC	Y	Y	N
Quinn *et al*.	2022	USA	Prospective	Unexplained; recurrent failure	Y	*	N	N	N	ICSI	ZyMōt ®	DGC	Y	Y	N
Tsuji *et al*.	2022	Japan	Prospective	severe male infertility was excluded	Y	*	Y	N	Y	ICSI	ZyMōt ®	DGC	Y	N	*
Buitrago *et al*.	2023	Spain	Prospective	Unexplained; recurrent failure. Severe male factor were excluded	Y	*	Y	N	Y	ICSI	*	DGC	Y	N	N
Mantravadi *et al*.	2023	India	Prospective	men with Smoking, Binge alcohol, varicocele excluded.	Y	Y	N	N	N	ICSI	ZyMōt ®	TESA / MACS / DGC	Y	Y	N
Ozaltin *et al*.	2023	Turkey	Prospective	*	Y	Y	N	N	N	ICSI	Fertile Chip®	DGC	Y	Y	N

* = not available

** = not applied; Y = yes; N = no; DGC = density gradient centrifugation; ICSI = intracytoplasmic sperm injection.

### Risk of bias and quality assessment of eligible studies

Through the application of the Newcastle-Ottawa scale for bias risk assessment (S4 Table), we determined the AHRQ evidence quality from the selected studies, detailed in Table 2. The 13 incorporated studies showcased commendable quality attributed to several reasons: they represented an inherently exposed cohort; they originated from the same community as the non-exposed cohort; they possessed reliable records; they lacked the outcome of interest at the study’s commencement; they maintained comparability between cohorts without confounding bias; their outcomes were assessed in both technical and binary manners; and they allowed ample duration for thorough evaluation.

**Table 2:** Quality assessment of eligible studies.

STUDY	YEAR	SELECTION				COMPARABILITY	OUTCOME			TOTAL (9/9)	QUALITY ASSESSMENT
		1	2	3	4	5	6	7	8		
Kalyan *et al*.	2019	★	★	★	★	★★	★	★		8/9	good quality
Yetkinel *et al*.	2019	★	★	★	★	★	★	★		7/9	good quality
Yildiz *et al*.	2019	★	★	★	★	★	★	★		7/9	good quality
Tavares *et al*.	2020	★	★	★	★	★	★	★		7/9	good quality
Anbari *et al*.	2021	★	★	★	★	★	★	★		7/9	good quality
Guler *et al*.	2021	★	★	★	★	★★	★	★		8/9	good quality
Leisinger *et al*.	2021	★	★	★	★	★★	★	★		8/9	good quality
Ozcan *et al*.	2021	★	★	★	★	★	★	★		7/9	good quality
Quinn *et al*.	2022	★	★	★	★	★	★	★		7/9	good quality
Tsuji *et al*.	2022	★	★	★	★	★★	★	★		8/9	good quality
Buitrago *et al*.	2023	★	★	★	★	★★	★	★		8/9	good quality
Mantravadi *et al*.	2023	★	★	★	★	★	★	★		7/9	good quality
Ozaltin *et al*.	2023	★	★	★	★	★	★	★		7/9	good quality

1) Representativeness of the exposed cohort; 2) Selection of the non exposed cohort; 3) Ascertainment of exposure; 4) Demonstration that outcome of interest was not present at start of study; 5) Comparability of cohorts on the basis of the design or analysis; 6) Assessment of outcome; 7) Was follow-up long enough for outcomes to occur; 8) Adequacy of follow up of cohorts.

Thresholds for converting the Newcastle-Ottawa scales to AHRQ standards (good, fair, and poor): Good quality: 3 or 4 stars in selection domain AND 1 or 2 stars in comparability domain AND 2 or 3 stars in outcome/exposure domain Fair quality: 2 stars in selection domain AND 1 or 2 stars in comparability domain AND 2 or 3 stars in outcome/exposure domain Poor quality: 0 or 1 star in selection domain OR 0 stars in comparability domain OR 0 or 1 stars in outcome/exposure domain

The utmost quality of evidence was given to studies that employed sibling oocytes for their experimental design [29–33]. Twelve of the studies adopted a prospective and randomized methodology [29–40], while a single study was retrospective and non-randomized [41]

No study was wholly omitted during the sensitivity analysis. Given that these are random effects, the scatter plot was solely utilized to evaluate the variance between studies, and none were removed based on their positioning (S1 Fig).

### Synthesis of results

This study assessed clinical pregnancy outcomes [29, 30, 34–41], miscarriage outcomes [29, 34, 36, 38–41], and other laboratory outcomes, including fertilization [29–32, 35–37, 39, 41, 42], cleavage [29, 31], total blastocysts [29–33, 39], high-quality blastocysts [29, 31, 37], and euploid blastocysts [30].

Of the 13 selected studies, six took place in Turkey [29, 31, 34, 35, 40, 41], three in the United States [30, 36, 38], one each in India [39], Spain [33], Japan [32], and Iran [37].

Two commercial microfluidic devices are mentioned in the studies: Fertile Chip® (Koek Biotechnology, Izmir/Turkey) [29, 31, 34, 35, 40, 41] and ZyMōt® (DxNow Inc., Gaithersburg/USA) [30, 32, 38, 39]. Notably, these are actually the same device, as a commercial agreement unified their branding, now both referred to as ZyMot®ICSI (Zymōt Fertility, Gaithersburg/USA) [43].

Concerning control techniques for sperm selection in the ICSI method, nine studies opted for density gradient centrifugation (DGC) [30–33, 35, 38–41], four employed the swim-up method [29, 30, 34, 37], one study used micro-TESE [36], and another used TESA and MACS [39].

Five of the studies used sibling oocytes for their experimental design [29–33], while eight adopted a different couples experimental approach [34–41].

Twelve studies had a prospective and randomized design [29–40], with only one being retrospective and non-randomized [41]. Sequential stimulation was used in one study [42] (Table 1).

Overall, we analyzed data from 2,223 infertile couples, involving 14,502 inseminated oocytes, 5,722 blastocysts, 598 embryo transfers, 868 clinical pregnancies, and 93 miscarriages (S5 Table).

We estimated miscarriage rates from studies that reported clinical pregnancies, ongoing pregnancies, and live births in three studies [34, 41], and in two additional studies [34, 41]. We reached out to 21 primary authors to request their raw data [29, 34, 35, 41, 44], receiving responses from three [29, 39, 41].

### Clinical results

#### Clinical pregnancy

**Fig 2:**
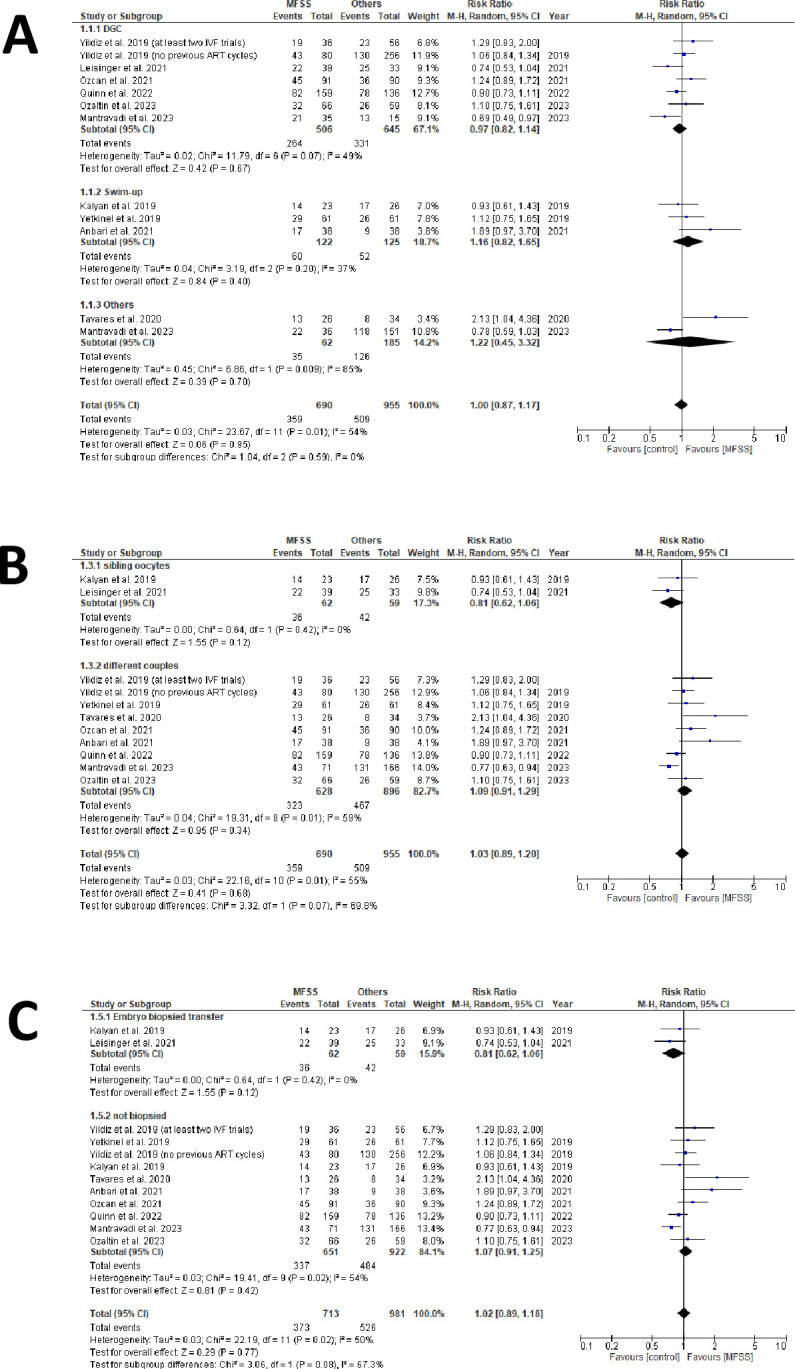
Clinical pregnancy outcomes forest plots. A) sperm sorter technique subgroups forest plot (MFSS *versus* DGC, swim-up, and others). The overall outcome suggests a slight improvement in clinical pregnancy results in favor of the control group, but without statistical significance. In subgroup analysis, it is possible to identify that this outcome is driven by the DGC sorter. No study was excluded based on the sensitivity test. B) allocation type subgroups forest plot (sibling oocytes *versus* different couples). The sensitivity test conducted did not identify any changes in the overall and subgroup outcomes. The overall outcome suggests a slight improvement in clinical pregnancy results in favor of MFSS, but without statistical significance. Subgroup results present conflicting findings. Sibling oocytes show favorable outcomes for the control group, while studies involving different couples show favorable outcomes for MFSS. No study was excluded in the sensitivity test; C) Euploidy subgroups forest plot (biopsied *versus* non biopsied). The overall outcome indicates a slight improvement in clinical pregnancy rates through the use of MFSS. For subgroup analysis, both studies suggest better results for the control groups. In non-biopsied embryos, most studies suggest a better outcome for MFSS. The sensitivity test suggests that the studies by Tavares *et al*. (2020) and Anbari *et al*. (2021) have an impact on the results and that, if excluded, the outcome would be favorable to the control groups.

Ten studies were chosen to evaluate clinical pregnancy outcomes [29, 30, 34–41]. In sum, we utilized data from 868 clinical pregnancies (359 from MFSS; 509 from control groups) derived from 1,646 embryo transfers (690 using MFSS; 956 using control methods) to produce forest plots (Fig 2 and S5 Table).

For the assessment of clinical pregnancy outcomes, we generated three forest plots using different approaches:

1. by sperm selection approach: MFSS *versus* DGC, swim-up or others (Fig 2A);
2. by allocation type: sibling oocytes *versus* different couples (Fig 2B); and
3. by embryo biopsy: transfer with euploidy selection *versus* none (Fig 2C).

#### Miscarriage

**Fig 3:**
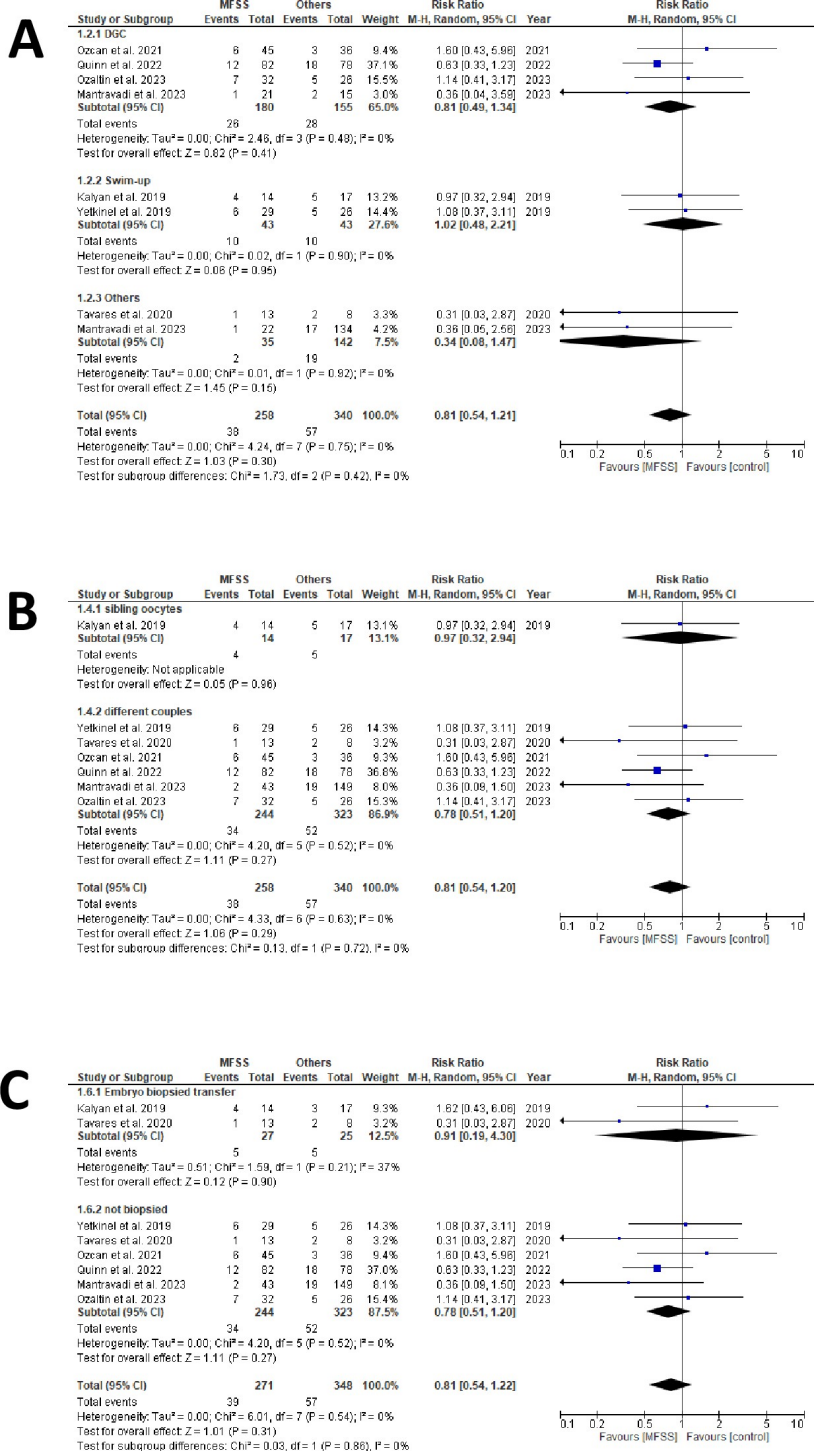
Miscarriage outcomes forest plots. A) sperm sorter technique subgroups forest plot (MFSS *versus* DGC, swim-up, and others). The overall outcome and subgroup results indicate a modest reduction in miscarriage rate through the utilization of MFSS, but without statistical significance. No study was excluded based on the sensitivity test; B) allocation type subgroups forest plot (sibling oocytes *versus* different couples). The overall outcome suggests a slight improvement in miscarriage rates when MFSS is used, but without statistical significance. For this evaluation, subgroup analysis was not possible as studies using sibling oocytes did not provide miscarriage rate data; C) euploidy subgroups forest plot (biopsied *versus* non biopsied). The overall outcome and subgroup results suggest lower miscarriage rates for the use of MFSS, but without statistical significance. No study was excluded in the sensitivity test.

Seven studies provided miscarriage data [29, 34, 35, 38–41], and among these, two contained data that required conversion [34, 41]. We created a forest plot based on data from 95 miscarriages (38 from MFSS; 57 from control groups) that occurred among 598 confirmed clinical pregnancies (258 using MFSS; 340 using control methods) (Fig 3 and S5 Table).

For the assessment of miscarriage outcomes, we generated three forest plots using different approaches:

4. by sperm selection approach: MFSS *versus* DGC, swim-up or others (Fig 3A);
5. by allocation type: sibling oocytes *versus* different couples (Fig 3B); and
6. by embryo biopsy: transfer with euploidy selection *versus* none (Fig 3C).

### Laboratory outcome results

#### Fertilization rate

**Fig 4:**
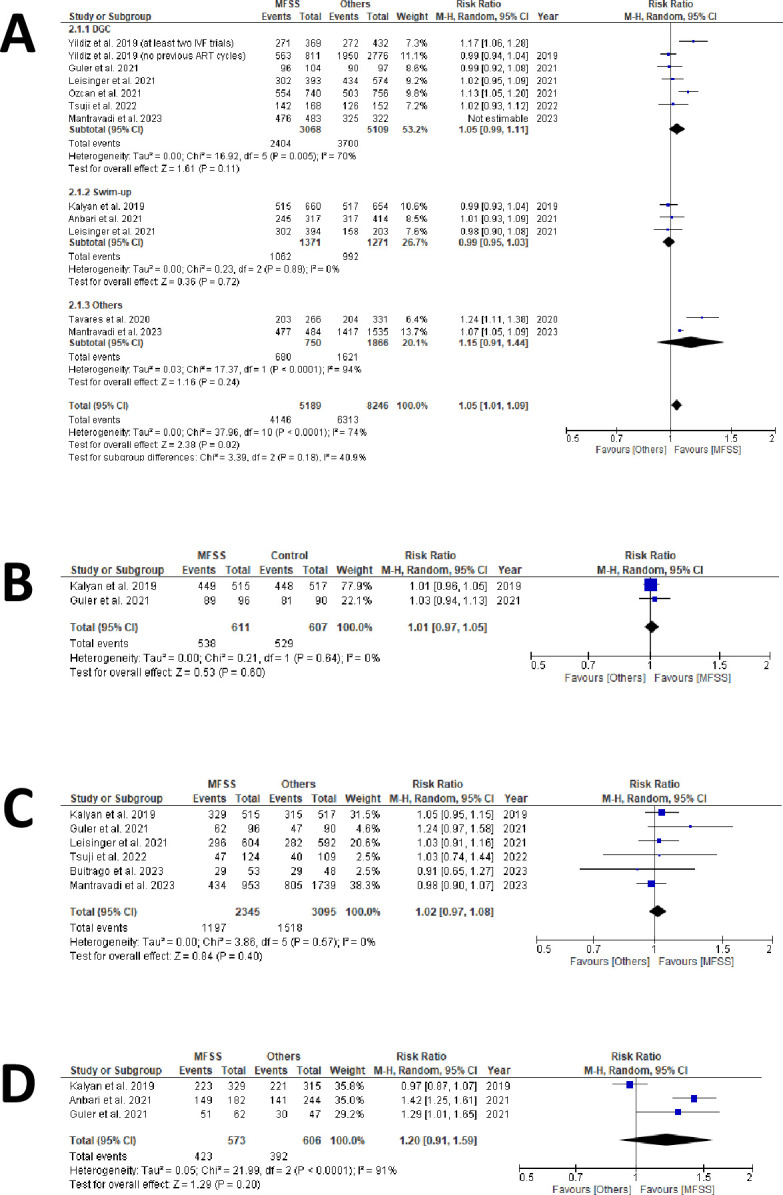
Laboratory outcomes of sperm sorter technique subgroups forest plots. A) fertilization outcomes forest plot; B) cleavage outcomes forest plot; C) total blastocyst forest plot; and D) blastocysts good/excelente forest plot.

Nine studies provided data on fertilized oocytes [29–32, 35–37, 39, 41]. We analyzed a forest plot based on data from 11,412 fertilized oocytes (5,099 from MFSS; 6,313 from control groups) out of 14,402 inseminated oocytes (6,156 using MFSS; 8,246 using control methods) (Fig 4A and S5 Table).

Additionally, we constructed another forest plot using data from 10,459 fertilized oocytes (4,146 from MFFS; 6,313 from control groups) out of 13,715 inseminated oocytes (5,328 using MFFS; 8,387 using control methods) (Fig 4A and S5 Table). This equates to fertilization rates of 75.3% for MFSS, and 71.7% for the control groups.

#### Cleavage rate

Only two studies provided raw data on cleavage rates [29, 31]. From our analysis, a forest plot was derived, encompassing data from 1,067 cleaved embryos (538 using MFSS; 529 from control groups) out of 1,218 inseminated oocytes (611 using MFSS; 607 using control methods) (Fig 4B and S5 Table). This data translates to cleavage rates of 88.05% when using MFSS, compared to 87.15% in the control groups.

#### Blastocysts total rate

Six studies provided raw data regarding blastocyst formation rates [29–33, 39]. Based on this data, we created a forest plot which included 2,715 blastocysts (1,197 from the MFSS group and 1,518 from the control group) derived from 5,475 inseminated oocytes (2,345 using MFSS and 3,095 from control methods). From the data, it can be inferred that the blastocyst formation rates, in relation to the total number of inseminated oocytes, were 51.04% for the MFSS group and 49.05% for the control group (Fig 4C and S5 Table).

#### Good/excellent blastocysts rate

Three studies assessed the quality of the produced blastocysts, categorizing them as either good or excellent [29, 31, 37]. From this data, we crafted a forest plot encompassing 815 blastocysts that were classified as either good or excellent. This total was derived from 423 blastocysts in the MFSS group and 392 blastocysts in the control group. These blastocysts originated from a total of 1,179 blastocysts, with 573 from the MFSS group and 606 from the control group. Based on this data, the rates for blastocysts considered as good or excellent were found to be 73.82% for the MFSS group and 64.69% for the control group. (Fig 4D and S5 Table).

### Evidence quality

We subjected our results to the GRADE methodology [27, 28]. Upon evaluation, the clinical evidence’s quality was consistently rated as "high" across all the subgroups, as detailed in S6 Table.

## Discussion

In line with our aim of investigating the potential benefits of microfluidic devices for sperm selection in enhancing clinical pregnancy and reducing miscarriage rates among couples undergoing ART treatments, our findings suggest modestly favorable outcomes, albeit lacking statistical significance. Similarly, our examination of laboratory parameters–encompassing fertilization, cleavage, and blastocyst formation rates–indicated marginal improvements, yet once again without achieving statistical significance.

We consciously decided to exclude studies that focused on seminal quality and sperm DNA damage as outcomes. Our rationale for this was rooted in the recognition that such indirect metrics yield divisive results and demand more standardized methodologies [45, 46]. In the light of risk of bias and evidence quality evaluations, two studies–Parrella et al. (2019) [47] and Kocur et al. (2022) [42]–were omitted from our analysis. This decision was informed by the fact that both studies leveraged successive stimulations from the same group as an experimental design for technique comparison. Future studies should ideally aim to address and eliminate this inherent bias, especially given the prevailing understanding that repeated stimulations tend to bolster success rates in ART [48, 49].

On the subject of the heterogeneity observed in our study, we believe that the uniformity of our statistical outcomes is justifiable. Such consistency can be attributed to the multifaceted and complex character of infertility [7, 50, 51], compounded by the variance in experimental designs and diverse study populations.

### Clinical pregnancy

For the clinical pregnancy outcome (Fig 2A), categorizing articles by sperm selection technique reveals DGC and MFSS results aligning closely with the null line. Though MFSS appears superior to swim-up, drawing firm conclusions is premature, especially given the sway of Anbari et al. (2021) [37] on this trend.

Other techniques produced mixed outcomes, not significantly altering the group’s overarching result. Consequently, we can deduce that current clinical sperm selection methods offer similar results. This aligns with findings from many studies we reviewed [7, 51].

Classifying studies by group allocation type in the experimental design (sibling oocytes versus different couples) (Fig 2B), we note those employing sibling oocytes showed better clinical pregnancy rates with standard sperm selection methods. But, caution is necessary due to the reliance on just two studies for this observation. A broader dataset would yield more definitive results [52]. Conversely, studies using different couples for comparison presented results mildly favoring MFSS, hovering near the null line.

Sorting studies by the use or non-use of embryonic biopsy for euploidy selection (Fig 2C), the two studies chosen for sibling oocytes also selected for euploid embryo transfer. This overlap potentially introduces bias, intertwining two distinct techniques’ evaluations. It’s worth noting that recent literature doesn’t conclusively support PGT-A’s benefits in success rates [53].

### Miscarriage

Regarding the miscarriage outcome (Fig 3A), when categorizing articles by sperm sorting techniques (MFSS versus DGC, swim-up, and others), we noted a slight advantage for MFSS, albeit not statistically significant. Notably, all studies intersected the null line, contrasting with the clinical pregnancy assessment.

For DGC, the outcome seemed influenced by one study [38]; however, its exclusion wouldn’t markedly change the overall outcome. The potential benefit of the method is further supported by two studies using unconventional sperm selection techniques: micro-TESE [36] and TESA and MACS [39].

When categorizing based on allocation type (sibling oocytes versus different couples) (Fig 3B), the limited data made it challenging to form conclusive inferences.

Sorting studies by the selection of euploidy for embryo transfer (Fig 3C), we encountered mixed results. Given the small sample size in this subgroup analysis [52] and the previously mentioned bias, it seems inappropriate to assert that both techniques have comparable outcomes.

For studies involving transfers of non-biopsied embryos, the data hint at a minor reduction in miscarriage rates with the use of MFSS, yet once more without statistical significance.

### Laboratory outcomes

Even though our primary focus was on assessing the techniques via the ultimate outcomes (clinical pregnancy and miscarriage), we utilized the available data to delve into laboratory outcomes.

For the nine studies presenting data on fertilization rates (Fig 4A), we observed a mild improvement with MFSS use, albeit not statistically significant. Interestingly, when swim-up was the control method, the rates appeared more favorable for this group. This observation aligns with the notion that techniques preserving the acrosomal cap might cause less DNA damage to sperm [54, 55], though this premise remains debated [56].

The evaluation of embryonic cleavage results (Fig 4B) was somewhat limited due to data availability from only two studies [29, 31]. However, with a sample size (N) surpassing 500, this data set can still be viewed as significant. The findings suggest that once sperm viability is established through fertilization, no discernible difference arises between MFSS and the control in terms of cleavage.

For total blastocyst formation (Fig 4C) and the derivation of good/excellent quality blastocysts (Fig 4D), the data leaned in favor of MFSS usage. Though not achieving statistical significance, these findings buttress the argument for enhanced outcomes when spermatozoa avoid rigorous procedures during ICSI selection [54].

### Evidence quality

Although the GRADE system rated the quality of evidence as high, it’s imperative to approach this information with a degree of skepticism.

While the risk of bias analysis and sensitivity testing ensured that no single study could sway the outcome, we advise caution in wholly trusting the final evidence. The elevated values seen for Chi² and I² serve as indicators of inconsistency [57, 58], urging a more nuanced interpretation of the findings.

## Conclusions

In summary, the data suggests a slight enhancement in ART outcomes when using microfluidic devices for sperm selection, albeit without any statistical significance. These findings highlight the pressing need for more consistent experimental designs in multicentric research settings to provide clearer answers.

In real-world clinical settings, other considerations like cost and time efficiency also play pivotal roles in selecting the sperm sorting method [59]. Our findings imply that such practical considerations can be prioritized, as there was no substantial difference in outcomes across the evaluated techniques. Moreover, we observed a gap in research, as there were no comparisons of MFSS outcomes with other emerging add-ons, such as the PICSI plate and technologies associated with hyaluronic acid binding.

## Strengths and limitations

The strengths of this study encompass a structured search strategy that allowed for the development of a reproducible systematic review and meta-analysis. The dichotomous nature of the data and the assurance of outcome absence during case selection provide confidence in the findings. Another strong point was the possibility of a relatively sizable sample size (N) and the potential for subsequent inclusion of new studies.

The main limitations lie in the heterogeneity of studies were inclusion/exclusion criteria and experimental design. The lack of statistical significance in the results could change with the incorporation of new studies. Once again, we emphasize the necessity for a robust multicenter study with a clearly delineated patient group framework.

## Supporting information

S1 Fig Eligible clinical outcomes studies dispersion plots. A) clinical pregnancy for sperm sporter technique dispersion plot; B) miscarriage for sperm sorter technique dispersion plot; C) clinical pregnancy for groups control dispersion plot; D) miscarriage for groups control dispersion plot; E) clinical pregnancy for embryo biopsy dispersion plot; F) miscarriage for embryo biopsy dispersion plot.
(PPTX)

S1 Table PRISMA checklist. (DOCX)

S2 Table Search strategies. (DOCX)

S3 Table Potential eligible studies. (DOCX)

S4 Table Risk of bias for eligible studies by Newcastle-Ottawa approach. (DOCX)

S5 Table Outcomes of eligible studies. (DOCX)

S6 Table GRADE evidence quality outcomes. (DOCX)
