# Supplementary material for: Does microfluidic sperm selection improve clinical pregnancy and miscarriage outcomes in assisted reproductive treatments? A systematic review and meta-analysis
Source: PLoS One. 2023 Nov 20;18(11):e0292891. doi: 10.1371/journal.pone.0292891 (PMC10659219; doi:10.1371/journal.pone.0292891)
Supplement: S1 Fig — A) clinical pregnancy for sperm sporter technique dispersion plot; B) miscarriage for sperm sorter technique dispersion plot; C) clinical pregnancy for groups control dispersion plot; D) miscarriage for groups control dispersion plot; E) clinical pregnancy for embryo biopsy dispersion plot; F) miscarriage for embryo biopsy dispersion plot. (PPTX) [file pone.0292891.s001.pptx]

## Slide 1
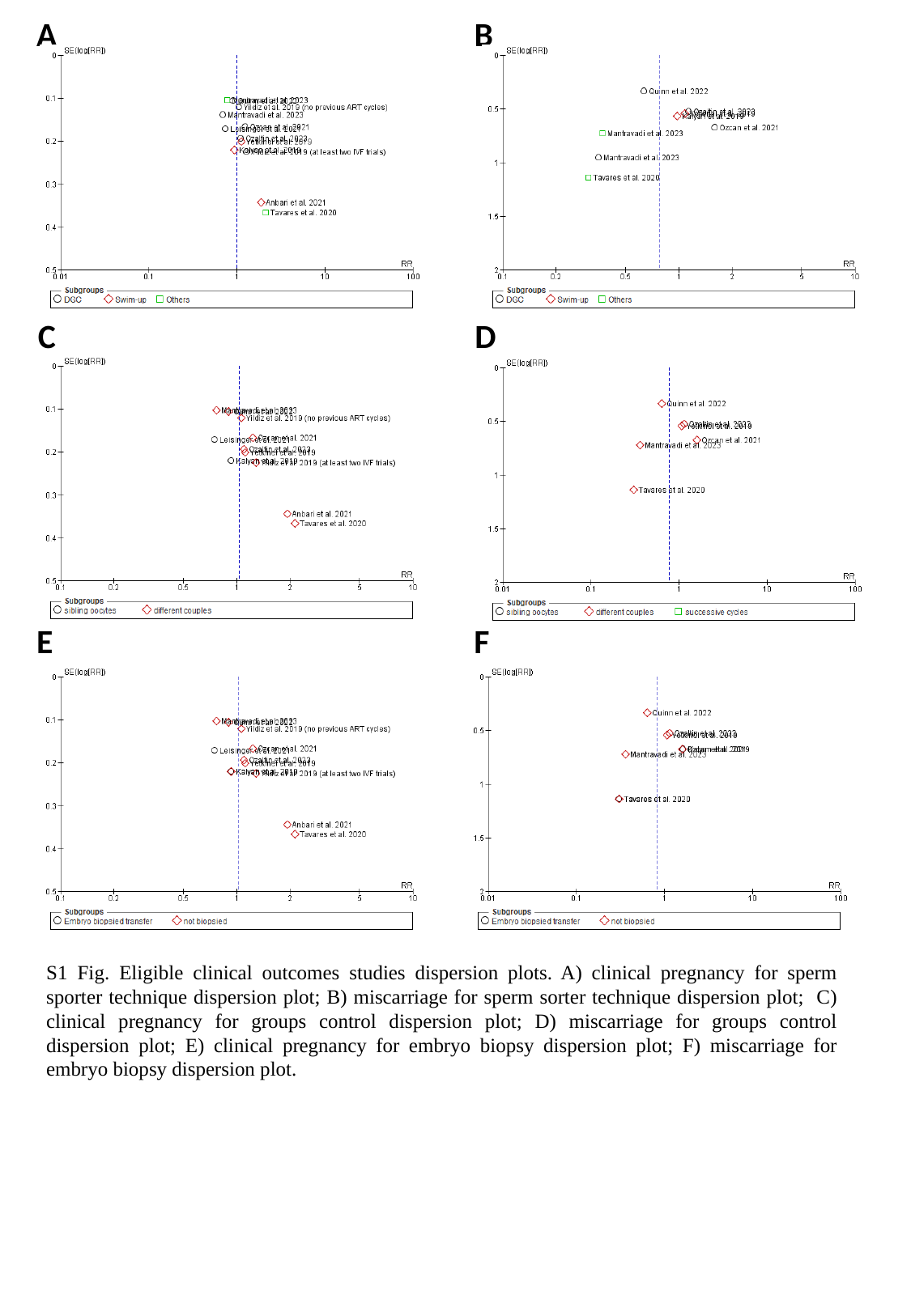

A
B
C
D
E
F
S1 Fig. Eligible clinical outcomes studies dispersion plots. A) clinical pregnancy for sperm sporter technique dispersion plot; B) miscarriage for sperm sorter technique dispersion plot; C) clinical pregnancy for groups control dispersion plot; D) miscarriage for groups control dispersion plot; E) clinical pregnancy for embryo biopsy dispersion plot; F) miscarriage for embryo biopsy dispersion plot.

## Slide 2
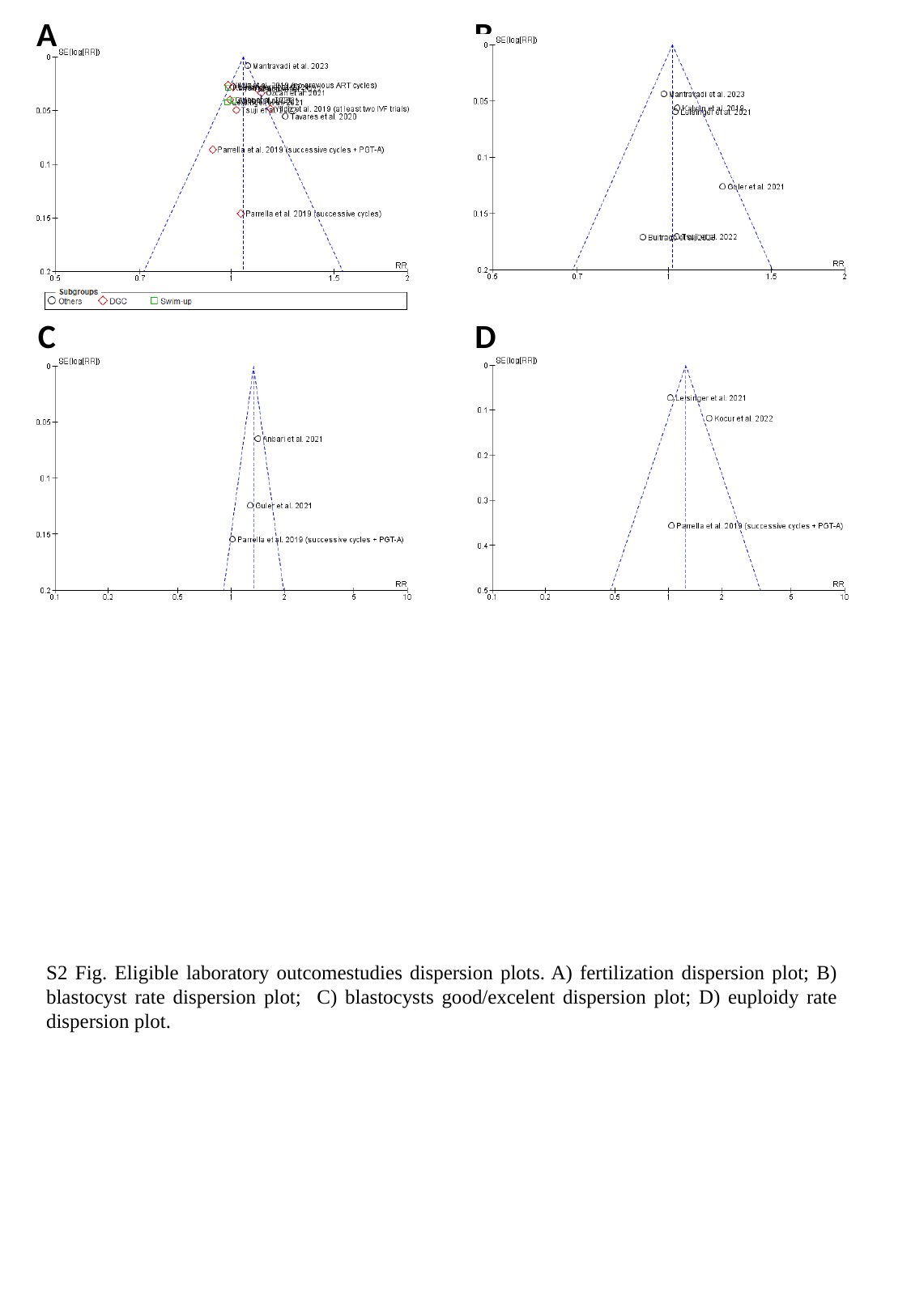

A
B
C
D
S2 Fig. Eligible laboratory outcomestudies dispersion plots. A) fertilization dispersion plot; B) blastocyst rate dispersion plot; C) blastocysts good/excelent dispersion plot; D) euploidy rate dispersion plot.
