# Supplementary material for: Does microfluidic sperm selection improve clinical pregnancy and miscarriage outcomes in assisted reproductive treatments? A systematic review and meta-analysis
Source: PLoS One. 2023 Nov 20;18(11):e0292891. doi: 10.1371/journal.pone.0292891 (PMC10659219; doi:10.1371/journal.pone.0292891)
Supplement: S2 Table — (DOCX) [file pone.0292891.s003.docx]

S2 Table. Search strategy.

| Pubmed  (2023-06-26) | ("microfluidics"[All Fields] OR "microfluidic"[All Fields] OR “MFSS”[All Fields]) AND (“semen”[All Fields] OR “seminal”[All Fields]; "sperm"[All Fields] OR "spermatozoa"[All Fields] OR “sperm sorter”[All Fields] OR “sperm selection”[All Fields]; “semen capacitation”[All Fields]) OR (“ICSI”[All Fields] OR “intracitoplasmatic sperm injection”[All Fields]) OR (“physiological intracytoplasmic sperm injection”[All Fields] OR “PICSI”[All Fields] OR “magnetic activated cell sorting”[All Fields] OR “MACS”[All Fields] OR “density gradient centrifugation”[All Fields] OR “DGC”[All Fields] OR “swim-up”[All Fields]) AND (“pregnancy”[All Fields] OR “miscarriage”[All Fields] OR “outcomes”[All Fields])  (9018 results) |
| --- | --- |
| National Institutes of Health - Clinical Trials (NIH)  (2023-06-26) | 1. Microfluidic sperm sorting (4) 2. Mfss (0) 3. Microfluidic sperm sorting chip (3) |
| LILACS  (2023-06-26) | 1. MFSS (1) 2. Microfluidic (11) 3. Microfluidis (4) 4. Microfluidic sperm (0) 5. Microfluidic semen (0) |
| CENTRAL  (2023-07-03) | 1. Microfluidic sperm (45) 2. Microfluidics (115) |
| Google Scholar  (2023-07-04) | 1. Microfluidic sperm sorting ivf (1830) 2. MFSS sperm (167) |
