# Supplementary material for: Does microfluidic sperm selection improve clinical pregnancy and miscarriage outcomes in assisted reproductive treatments? A systematic review and meta-analysis
Source: PLoS One. 2023 Nov 20;18(11):e0292891. doi: 10.1371/journal.pone.0292891 (PMC10659219; doi:10.1371/journal.pone.0292891)
Supplement: S3 Table — (DOCX) [file pone.0292891.s004.docx]

| S3 Table. Potential eligible studies. | | | | | | | | |
| --- | --- | --- | --- | --- | --- | --- | --- | --- |
| **Author** | **Year** | **Country** | **Reason for exclusion** | **Type of information** | | **Type of seminal treatment** | | **Sibling oocytes** |
|  |  |  |  | **Laboratory outcomes** | **Clinical outcomes** | **Type of MFSS** | **Type of control** |  |
| Dogan *et al.* | 2017 | Turkey | clinical trial ongoing | N | Y | Sperm Chip | swim-up | N |
| Mitchell *et al.* | 2017 | USA | clinical trial ongoing | Y | Y | Fertile Plus® | DGC | N |
| Pabuccu *et al.* | 2018 | Turkey | abstract of meetings | Y | N | * | * | N |
| Basar *et al.* | 2019 | Turkey | no response to our contact | Y | N | Fertile Plus® | DGC | N |
| Gode *et al.* | 2019 | Turkey | IUI | N | Y | Fertile Plus® | DGC | N |
| Parrella *et al.* | 2019 | USA | successive stimulus in the same group | Y | Y | ZyMōt™ | DGC | N |
| Khan *et al.* | 2020 | India | abstract of meetings | Y | Y | * | DGC | N |
| Matsunaga *et al.* | 2020 | Japan | no response to our contact | Y | N | ZyMōt™ | DGC | N |
| Palmerola *et al.* | 2020 | USA | abstract of meetings | N | Y | * | CDG | N |
| Pavlovic *et al.* | 2020 | USA | no response to our contact | Y | Y | * | DGC | N |
| Garcillan *et al.* | 2021 | Spain | abstract of meetings | Y | N | * | MACS | Y |
| González-Ravina *et al.* | 2021 | Spain | abstract of meetings | Y | Y | * | MACS | Y |
| Higashiyama *et al.* | 2021 | Japan | abstract of meetings | Y | N | ZyMōt™ | DGC | Y |
| Kant *et al.* | 2021 | India | abstract of meetings | Y | Y | * | PICSI | N |
| Keating *et al.* | 2021 | USA | abstract of meetings | Y | Y | * | DGC | N |
| Keskin *et al.* | 2021 | Turkey | no response to our contact | Y | Y | * | DGC | N |
| Lee *et al.* | 2021 | USA | abstract of meetings | Y | * | * | DGC | N |
| Lynn *et al.* | 2021 | USA | abstract of meetings | Y | N | ZyMōt™ | DGC | N |
| Morishita *et al.* | 2021 | Japan | abstract of meetings | Y | Y | ZyMōt™ | DGC | N |
| Patel *et al.* | 2021 | USA | abstract of meetings | Y | N | ZyMōt™ | DGC | N |
| Pujol *et al.* | 2021 | Spain | study has no control group | Y | Y | ZyMōt™ | * | N |
| Robles e*t al.* | 2021 | USA | abstract of meetings | Y | Y | ZyMōt™ | DGC | N |
| Schiewe *et al.* | 2021 | USA | no response to our contact | Y | N | ZyMōt™ | DGC / TESA | N |
| Sipahi *et al.* | 2021 | Turkey | IUI | N | Y | * | * | N |
| Vergueiro *et al.* | 2021 | Brazil | abstract of meetings | Y | N | ZyMōt™ | swim-up | N |
| Engmann *et al.* | 2021 | USA | clinical trial ongoing | Y | N | ZyMōt™ | DGC | Y |
| Fritz *et al.* | 2021 | USA | clinical trial ongoing | Y | N | ZyMōt™ | DGC | Y |
| Higashiyama *et al.* | 2021 | Japan | no response to our contact | Y | Y | * | DGC | Y |
| Lara-Cerrillo *et al.* | 2021 | Spain | no response to our contact | Y | Y | ZyMōt™ (Fertile Chip®) | DGC / swim-up | N |
| Lee *et al.* | 2021 | USA | no response to our contact | Y | Y | * | DGC | N |
| Patel *et a.* | 2021 | USA | no response to our contact | Y | Y | * | DGC | N |
| Vergueiro *et al.* | 2021 | Brazil | no response to our contact | Y | Y | ZyMōt™ (Fertile Chip®) | swim-up | N |
| Acet *et al.* | 2022 | Turkey | no response to our contact | N | Y | * | swim-up | N |
| Aydin *et al.* | 2022 | Turkey | no response to our contact | Y | Y | Fertile Chip® | swim-up | N |
| Berton *et al.* | 2022 | Brazil | abstract of meetings | Y | Y | * | DGC + swim-up | N |
| Godiwala *et al.* | 2022 | USA | no response to our contact | Y | Y | ZyMōt™ | DGC | Y |
| Keskin *et al.* | 2022 | Turkey | no response to our contact | Y | Y | * | DGC | N |
| Kocur *et al.* | 2022 | USA | successive stimulus in the same group | Y | Y | ZyMōt™ | DGC | N |
| Mirsanei *et al.* | 2022 | Iran | no response to our contact | Y | N | * | DGC | N |
| Srinivas *et al.* | 2022 | India | egg donation | N | Y | * | DGC | N |
| Akram et al. | 2023 | Canada | Abstract | Y | Y | ZyMōt™ | TESA | N |
| Balakaier *et al.* | 2023 | Canada | no response to our contact | Y | Y | ZyMōt™ | DGC | N |
| Budak *et al.* | 2023 | Turkey | no response to our contact | Y | Y | * | swim-up | N |
| Debnah *et al.* | 2023 | India | no response to our contact | Y | N | * | TESA / MACS / DGC | N |
| Huniadi *et al.* | 2023 | Romania | abstract of meetings | Y | Y | ZyMōt™ | swim-up | N |
| Moutos *et al.* | 2023 | USA | IUI | N | Y | ZyMōt™ (Fertile Chip®) | DGC | N |
| Zaha *et al.* | 2023 | Romania | no response to our contact | Y | Y | ZyMōt™ | DGC | N |
| Y = yes; N = no; * = not available; DGC = density gradient centrifugation; MACS = magnetic-activated cell sorting. | | | | | | | | |
