# Supplementary material for: Does microfluidic sperm selection improve clinical pregnancy and miscarriage outcomes in assisted reproductive treatments? A systematic review and meta-analysis
Source: PLoS One. 2023 Nov 20;18(11):e0292891. doi: 10.1371/journal.pone.0292891 (PMC10659219; doi:10.1371/journal.pone.0292891)
Supplement: S4 Table — (DOCX) [file pone.0292891.s005.docx]

S4 Table. Risk of bias for eligible studies by Newcastle-Ottawa approach.

| DIMENSIONS/ AUTHORS | Kalyan *et al.* (2019) | Yetkinel *et al.* (2019) | | Yildiz *et al*. (2019) | | Tavares *et al*. (2020) | | Anbari *et al.* (2021) | Guler *et al.* (2021) | | Leisinger *et al.* (2021) | | Ozcan *et al.* (2021) | | Quinn *et al.* (2022) | Tsuji *et al.* (2022) | | Buitrago *et al.* (2023) | | Mantravadi *et al.* (2023) | | Ozaltin *et al.* (2023) | |  |  |  |  |  |  |  |
| --- | --- | --- | --- | --- | --- | --- | --- | --- | --- | --- | --- | --- | --- | --- | --- | --- | --- | --- | --- | --- | --- | --- | --- | --- | --- | --- | --- | --- | --- | --- |
| **Selection** |  |  | |  | |  | |  |  | |  | |  | |  |  | |  | |  | |  | |  |  |  |  |  |  |  |
| 1) Representativeness of the exposed cohort | a | a | | a | | b | | a | b | | a | | b | | a | a | | a | | b | | b | |  |  |  |  |  |  |  |
| 2) Selection of the non exposed cohort | a | a | | a | | a | | a | a | | a | | a | | a | a | | a | | a | | a | |  |  |  |  |  |  |  |
| 3) Ascertainment of exposure | a | a | | a | | a | | a | a | | a | | a | | a | a | | a | | a | | a | |  |  |  |  |  |  |  |
| 4) Demonstration that outcome of interest was not present at start of study | a | a | | a | | a | | a | a | | a | | a | | a | a | | a | | a | | a | |  |  |  |  |  |  |  |
| **Comparability** |  |  | |  | |  | |  |  | |  | |  | |  |  | |  | |  | |  | |  |  |  |  |  |  |  |
| 5) Comparability of cohorts on the basis of the design or analysis | a, b | a | | a | | a | | a | a,b | | a,b | | a | | a | a,b | | a,b | | a | | a | |  |  |  |  |  |  |  |
| **Outcome** |  |  | |  | |  | |  |  | |  | |  | |  |  | |  | |  | |  | |  |  |  |  |  |  |  |
| 6) Assessment of outcome | b | b | | b | | b | | b | b | | b | | b | | b | b | | b | | b | | b | |  |  |  |  |  |  |  |
| 7) Was follow-up long enough for outcomes to occur | a | a | | a | | a | | a | a | | a | | a | | a | a | | a | | a | | a | |  |  |  |  |  |  |  |
| 8) Adequacy of follow up of cohorts | d | d | | d | | d | | d | d | | d | | d | | d | d | | d | | d | | d | |  |  |  |  |  |  |  |
| Legends: | | |  | |  | |  | | |  | |  | |  | | |  | |  | |  | | | |  |  |  |  |  |  |
| 1) Representativeness of the exposed cohort: a) truly representative of the average of infertile couples submitted to ART in the community; b) somewhat representative of the average of infertile couples submitted to ART in the community; c) selected group of users eg nurses, volunteers; d) no description of the derivation of the cohort. | | | | | | | | | | | | | | | | | | | | | | |  |  |  |  |  |  |  |  |
| 2) Selection of the non exposed cohort: a) drawn from the same community as the exposed cohort; b) drawn from a different source; c) no description of the derivation of the non exposed cohort. | | | | | | | | | | | | | | | | | | | | | | |  |  |  |  |  |  |  |  |
| 3) Ascertainment of exposure: a) secure record (eg surgical records); b) structured interview; c) written self report; d) no description. | | | | | | | | | | | | | | | | | | | | | | |  |  |  |  |  |  |  |  |
| 4) Demonstration that outcome of interest was not present at start of study: a) yes; b) no. | | | | | | | | | | | | | | | | | | | | | | |  |  |  |  |  |  |  |  |
| 5) Comparability of cohorts on the basis of the design or analysis: a) study controls for clinical pregnacy and miscarriage rates; b) b) study controls for any additional factor (laboratory outcomes as fertilization, cleavage and blastocyst rates). | | | | | | | | | | | | | | | | | | | | | | |  |  |  |  |  |  |  |  |
| 6) Assessment of outcome: a) independent blind assessment; b) record linkage; c) self report; d) no description. We created the 'e' option for explain the use for criteria grades, but without blinding. | | | | | | | | | | | | | | | | | | | | | | |  |  |  |  |  |  |  |  |
| 7) Was follow-up long enough for outcomes to occur: a) yes (select an adequate follow up period for outcome of interest); b) no. | | | | | | | | | | | | | | | | | | | | | | |  |  |  |  |  |  |  |  |
| 8) Adequacy of follow up of cohorts: a) complete follow up - all subjects accounted for; b) subjects lost to follow up unlikely to introduce bias - small number lost - > 80 % follow up, or description provided of those lost); c) follow up rate < 79 % and no description of those lost; d) no statement. | | | | | | | | | | | | | | | | | | | | | | |  |  |  |  |  |  |  |  |
