# Supplementary material for: Does microfluidic sperm selection improve clinical pregnancy and miscarriage outcomes in assisted reproductive treatments? A systematic review and meta-analysis
Source: PLoS One. 2023 Nov 20;18(11):e0292891. doi: 10.1371/journal.pone.0292891 (PMC10659219; doi:10.1371/journal.pone.0292891)
Supplement: S5 Table — (DOCX) [file pone.0292891.s006.docx]

S5 Table. Outcomes of eligible studies.

| **Study identification** | | **Participants data** | | | **Laboratory outcomes** | | | | | | | | | | | | | | | | | | | | **Clinical outcomes** | | | | | | | | **Conclusion in study** |
| --- | --- | --- | --- | --- | --- | --- | --- | --- | --- | --- | --- | --- | --- | --- | --- | --- | --- | --- | --- | --- | --- | --- | --- | --- | --- | --- | --- | --- | --- | --- | --- | --- | --- |
|  |  |  |  |  | **Fertilized oocytes** | | | | **Cleaved embryos** | | | | **Blastocysts total (blastocyst/fertilized)** | | | | **Blastocysts (good/excellent)** | | | | **Euploidy** | | | | **Clinical pregnancy (implantation/transfered)** | | | | **Miscarriage (loss/pregnancy)** | | | |  |
|  |  |  |  |  | **MFSS** | | **Contr.** | | **MFSS** | | **Contr.** | | **MFSS** | | **Contr.** | | **MFSS** | | **Contr.** | | **MFSS** | | **Contr.** | | **MFSS** | | **Contr.** | | **MFSS** | | **Contr.** | |  |
| **Author** | **Year** | **Infertility couples** | **MFSS** | **Control** | **E** | **T** | **E** | **T** | **E** | **T** | **E** | **T** | **E** | **T** | **E** | **T** | **E** | **T** | **E** | **T** | **E** | **T** | **E** | **T** | **E** | **T** | **E** | **T** | **E** | **T** | **E** | **T** |  |
| Kalyan et al. | 2019 | 81 | 81 | 81 | 515 | 660 | 517 | 654 | 449 | 515 | 448 | 517 | 329 | 515 | 315 | 517 | 223 | 329 | 221 | 315 | * | * | * | * | 14 | 23 | 17 | 26 | 4 | 14 | 5 | 17 | no significant improvement |
| Yetkinel et al. | 2019 | 122 | 61 | 61 | * | * | * | * | * | * | * | * | * | * | * | * | * | * | * | * | * | * | * | * | 29 | 61 | 26 | 61 | 6 | 29 | 5 | 26 | no significant improvement |
| Yildiz et al. | 2019 | 336 | 80 | 256 | 563 | 811 | 1950 | 2776 | * | * | * | * | * | * | * | * | * | * | * | * | * | * | * | * | 43 | 80 | 130 | 256 | * | * | * | * | no significant improvement |
| Yildiz et al. | 2019 | 92 | 36 | 56 | 271 | 369 | 272 | 432 | * | * | * | * | * | * | * | * | * | * | * | * | * | * | * | * | 19 | 36 | 23 | 56 | * | * | * | * | significant improvement in fertilization rate |
| Tavares et al. | 2020 | 44 | 22 | 22 | 203 | 266 | 204 | 331 | * | * | * | * | * | * | * | * | * | * | * | * | * | * | * | * | 13 | 26 | 8 | 34 | 1 | 13 | 2 | 8 | significant improvement in all parameters |
| Anbari et al. | 2021 | 95 | 45 | 50 | 245 | 317 | 317 | 414 | * | * | * | * | * | * | * | * | 149 | 182 | 141 | 244 | * | * | * | * | 17 | 38 | 9 | 39 | * | * | * | * | significant improvement in blastocyst quality and clinical pregnancy |
| Guler et al. | 2021 | 22 | 22 | 22 | 96 | 104 | 90 | 97 | 89 | 96 | 81 | 90 | 62 | 96 | 47 | 90 | 51 | 62 | 30 | 47 | ** | ** | ** | ** | ** | ** | ** | ** | ** | ** | ** | ** | significant improvement in blastocyst quality |
| Leisinger et al. | [2021](https://www.mdpi.com/2673-3897/2/3/13) | 63 | 63 | 63 | 604 | 787 | 434 | 574 | * | * | * | * | 296 | 604 | 190 | 434 | * | * | * | * | 165 | 283 | 99 | 185 | 22 | 39 | 25 | 33 | * | * | * | * | no significant improvement |
| Leisinger et al. | [2021](https://www.mdpi.com/2673-3897/2/3/13) | 23 | 23 | 23 | 604 | 787 | 158 | 203 | * | * | * | * | 296 | 604 | 92 | 158 | * | * | * | * | 165 | 283 | 52 | 80 | * | * | * | * | * | * | * | * | significant improvement in blastocyst rate |
| Ozcan et al. | 2021 | 181 | 91 | 90 | 554 | 740 | 503 | 756 | * | * | * | * | * | * | * | * | * | * | * | * | * | * | * | * | 45 | 91 | 36 | 90 | 6 | 45 | 3 | 36 | no significant improvement |
| Quinn et al. | 2022 | 297 | 157 | 140 | *** | *** | *** | *** | *** | *** | *** | *** | *** | *** | *** | *** | *** | *** | *** | *** | ** | ** | ** | ** | 82 | 159 | 78 | 136 | 12 | 82 | 18 | 78 | no significant improvement |
| Tsuji et al. | 2022 | 20 | 20 | 20 | 142 | 168 | 126 | 152 | * | * | * | * | 47 | 124 | 40 | 109 | * | * | * | * | ** | ** | ** | ** | ** | ** | ** | ** | ** | ** | ** | ** | no significant improvement |
| Buitrago et al. | 2023 | 29 | 29 | 29 | * | 139 | * | 141 | * | * | * | * | 29 | 53 | 29 | 48 | * | * | * | * | * | * | * | * | * | * | * | * | * | * | * | * | no significant improvement |
| Mantravadi et al. | 2023 | 245 | 116 | 129 | 953 | 967 | 524 | 616 | * | * | * | * | 434 | 953 | 240 | 524 | * | * | * | * | * | * | * | * | 43 | 71 | 69 | 86 | 2 | 43 | 10 | 69 | no significant improvement |
| Mantravadi et al. | 2023 | 208 | 116 | 92 | 953 | 967 | 893 | 919 | * | * | * | * | 434 | 953 | 413 | 893 | * | * | * | * | * | * | * | * | 43 | 71 | 49 | 65 | 2 | 43 | 7 | 65 | no significant improvement |
| Mantravadi et al. | 2023 | 152 | 116 | 36 | 953 | 967 | 325 | 322 | * | * | * | * | 434 | 953 | 152 | 322 | * | * | * | * | * | * | * | * | 43 | 71 | 13 | 15 | 2 | 43 | 2 | 15 | no significant improvement |
| Ozaltin et al. | 2023 | 213 | 102 | 111 | *** | *** | *** | *** | *** | *** | *** | *** | *** | *** | *** | *** | *** | *** | *** | *** | *** | *** | *** | *** | 32 | 66 | 26 | 59 | 7 | 32 | 5 | 26 | significant improvement in blastocyst quality |
| **Total** | | | | | **6656** | **8049** | **6313** | **8387** | **538** | **611** | **529** | **607** | **2361** | **4855** | **1518** | **3095** | **423** | **573** | **392** | **606** | **330** | **566** | **151** | **265** | **445** | **832** | **509** | **956** | **42** | **344** | **57** | **340** | **+** |
| Legends: * = not available; ** = not applied; Y = yes; N = no; DGC = density gradient centrifugation; ICSI = intracytoplasmic sperm injection | | | | | | | | | | | | | | | | | | Contr = control, E = events, T = total; * = not available; ** = not applied; *** = data in percentual or median values | | | | | | | | | | | | | | | |
