# Supplementary material for: Does microfluidic sperm selection improve clinical pregnancy and miscarriage outcomes in assisted reproductive treatments? A systematic review and meta-analysis
Source: PLoS One. 2023 Nov 20;18(11):e0292891. doi: 10.1371/journal.pone.0292891 (PMC10659219; doi:10.1371/journal.pone.0292891)
Supplement: S6 Table — (DOCX) [file pone.0292891.s007.docx]

S6 Table. GRADE evidence quality clinical outcomes.

**Question:** Clinical outcomes compared to control sperm sorting in assisted reproductive treatments

| **Certainty assessment** | | | | | | | **№ of patients** | | **Effect** | | **Certainty** | **Importance** |
| --- | --- | --- | --- | --- | --- | --- | --- | --- | --- | --- | --- | --- |
| **№ of studies** | **Study design** | **Risk of bias** | **Inconsistency** | **Indirectness** | **Imprecision** | **Other considerations** | **Clinical outcomes** | **placebo** | **Relative (95% CI)** | **Absolute (95% CI)** |  |  |
| **Clinical pregnancy - sperm sorter technique** | | | | | | | | | | | | |
| 11 | observational studies | not serious | not serious | not serious | not serious | all plausible residual confounding would suggest spurious effect, while no effect was observed | 359/690 (52.0%) | 509/955 (53.3%) | **RR 1.00** (0.87 to 1.17) | **0 fewer per 1.000** (from 69 fewer to 91 more) | ⨁⨁⨁⨁ High |  |
| **Clinical pregnancy - sperm sorter technique - DGC** | | | | | | | | | | | | |
| 7 | observational studies | not serious | not serious | not serious | not serious | all plausible residual confounding would suggest spurious effect, while no effect was observed | 264/506 (52.2%) | 331/645 (51.3%) | **RR 0.97** (0.82 to 1.14) | **15 fewer per 1.000** (from 92 fewer to 72 more) | ⨁⨁⨁⨁ High |  |
| **Clinical pregnancy - sperm sorter technique - Swim-up** | | | | | | | | | | | | |
| 3 | observational studies | not serious | not serious | not serious | not serious | all plausible residual confounding would suggest spurious effect, while no effect was observed | 60/122 (49.2%) | 52/125 (41.6%) | **RR 1.16** (0.82 to 1.65) | **67 more per 1.000** (from 75 fewer to 270 more) | ⨁⨁⨁⨁ High |  |
| **Clinical pregnancy - sperm sorter technique - Others** | | | | | | | | | | | | |
| 2 | observational studies | not serious | not serious | not serious | not serious | all plausible residual confounding would suggest spurious effect, while no effect was observed | 35/62 (56.5%) | 126/185 (68.1%) | **RR 1.22** (0.45 to 3.32) | **150 more per 1.000** (from 375 fewer to 1.000 more) | ⨁⨁⨁⨁ High |  |
| **Miscarriage - sperm sorter technique** | | | | | | | | | | | | |
| 7 | observational studies | not serious | not serious | not serious | not serious | all plausible residual confounding would suggest spurious effect, while no effect was observed | 38/258 (14.7%) | 57/340 (16.8%) | **RR 0.81** (0.54 to 1.21) | **32 fewer per 1.000** (from 77 fewer to 35 more) | ⨁⨁⨁⨁ High |  |
| **Miscarriage - sperm sorter technique - DGC** | | | | | | | | | | | | |
| 4 | observational studies | not serious | not serious | not serious | not serious | all plausible residual confounding would suggest spurious effect, while no effect was observed | 26/180 (14.4%) | 28/155 (18.1%) | **RR 0.81** (0.49 to 1.34) | **34 fewer per 1.000** (from 92 fewer to 61 more) | ⨁⨁⨁⨁ High |  |
| **Miscarriage - sperm sorter technique - Swim-up** | | | | | | | | | | | | |
| 2 | observational studies | not serious | not serious | not serious | not serious | all plausible residual confounding would suggest spurious effect, while no effect was observed | 10/43 (23.3%) | 10/43 (23.3%) | **RR 1.02** (0.48 to 2.21) | **5 more per 1.000** (from 121 fewer to 281 more) | ⨁⨁⨁⨁ High |  |
| **Miscarriage - sperm sorter technique - Others** | | | | | | | | | | | | |
| 2 | observational studies | not serious | not serious | not serious | not serious | all plausible residual confounding would suggest spurious effect, while no effect was observed | 2/35 (5.7%) | 19/142 (13.4%) | **RR 0.34** (0.08 to 1.47) | **88 fewer per 1.000** (from 123 fewer to 63 more) | ⨁⨁⨁⨁ High |  |
| **Clinical pregnancy - groups control** | | | | | | | | | | | | |
| 11 | observational studies | not serious | not serious | not serious | not serious | all plausible residual confounding would suggest spurious effect, while no effect was observed | 359/690 (52.0%) | 509/955 (53.3%) | **RR 1.03** (0.89 to 1.20) | **16 more per 1.000** (from 59 fewer to 107 more) | ⨁⨁⨁⨁ High |  |
| **Clinical pregnancy - groups control - sibling oocytes** | | | | | | | | | | | | |
| 2 | observational studies | not serious | not serious | not serious | not serious | all plausible residual confounding would suggest spurious effect, while no effect was observed | 36/62 (58.1%) | 42/59 (71.2%) | **RR 0.81** (0.62 to 1.06) | **135 fewer per 1.000** (from 271 fewer to 43 more) | ⨁⨁⨁⨁ High |  |
| **Clinical pregnancy - groups control - different couples** | | | | | | | | | | | | |
| 9 | observational studies | not serious | not serious | not serious | not serious | all plausible residual confounding would suggest spurious effect, while no effect was observed | 323/628 (51.4%) | 467/896 (52.1%) | **RR 1.09** (0.91 to 1.29) | **47 more per 1.000** (from 47 fewer to 151 more) | ⨁⨁⨁⨁ High |  |
| **Miscarriage - groups control** | | | | | | | | | | | | |
| 7 | observational studies | not serious | not serious | not serious | not serious | all plausible residual confounding would suggest spurious effect, while no effect was observed | 38/258 (14.7%) | 57/340 (16.8%) | **RR 0.81** (0.54 to 1.20) | **32 fewer per 1.000** (from 77 fewer to 34 more) | ⨁⨁⨁⨁ High |  |
| **Miscarriage - groups control - sibling oocytes** | | | | | | | | | | | | |
| 1 | observational studies | not serious | not serious | not serious | not serious | all plausible residual confounding would suggest spurious effect, while no effect was observed | 4/14 (28.6%) | 5/17 (29.4%) | **RR 0.97** (0.32 to 2.94) | **9 fewer per 1.000** (from 200 fewer to 571 more) | ⨁⨁⨁⨁ High |  |
| **Miscarriage - groups control - different couples** | | | | | | | | | | | | |
| 6 | observational studies | not serious | not serious | not serious | not serious | all plausible residual confounding would suggest spurious effect, while no effect was observed | 34/244 (13.9%) | 52/323 (16.1%) | **RR 0.78** (0.51 to 1.20) | **35 fewer per 1.000** (from 79 fewer to 32 more) | ⨁⨁⨁⨁ High |  |
| **Clinical pregnancy - Embryo biopsied transfer** | | | | | | | | | | | | |
| 11 | observational studies | not serious | not serious | not serious | not serious | all plausible residual confounding would suggest spurious effect, while no effect was observed | 373/713 (52.3%) | 526/981 (53.6%) | **RR 1.02** (0.89 to 1.18) | **11 more per 1.000** (from 59 fewer to 97 more) | ⨁⨁⨁⨁ High |  |
| **Clinical pregnancy - Embryo biopsied transfer - Embryo biopsied transfer** | | | | | | | | | | | | |
| 2 | observational studies | not serious | not serious | not serious | not serious | all plausible residual confounding would suggest spurious effect, while no effect was observed | 36/62 (58.1%) | 42/59 (71.2%) | **RR 0.81** (0.62 to 1.06) | **135 fewer per 1.000** (from 271 fewer to 43 more) | ⨁⨁⨁⨁ High |  |
| **Clinical pregnancy - Embryo biopsied transfer - not biopsied** | | | | | | | | | | | | |
| 10 | observational studies | not serious | not serious | not serious | not serious | all plausible residual confounding would suggest spurious effect, while no effect was observed | 337/651 (51.8%) | 484/922 (52.5%) | **RR 1.07** (0.91 to 1.25) | **37 more per 1.000** (from 47 fewer to 131 more) | ⨁⨁⨁⨁ High |  |
| **Miscarriage - embryo biopsied transfer** | | | | | | | | | | | | |
| 7 | observational studies | not serious | not serious | not serious | not serious | all plausible residual confounding would suggest spurious effect, while no effect was observed | 39/271 (14.4%) | 57/348 (16.4%) | **RR 0.81** (0.54 to 1.22) | **31 fewer per 1.000** (from 75 fewer to 36 more) | ⨁⨁⨁⨁ High |  |
| **Miscarriage - embryo biopsied transfer - Embryo biopsied transfer** | | | | | | | | | | | | |
| 2 | observational studies | not serious | not serious | not serious | not serious | all plausible residual confounding would suggest spurious effect, while no effect was observed | 5/27 (18.5%) | 5/25 (20.0%) | **RR 0.91** (0.19 to 4.30) | **18 fewer per 1.000** (from 162 fewer to 660 more) | ⨁⨁⨁⨁ High |  |
| **Miscarriage - embryo biopsied transfer - not biopsied** | | | | | | | | | | | | |
| 6 | observational studies | not serious | not serious | not serious | not serious | all plausible residual confounding would suggest spurious effect, while no effect was observed | 34/244 (13.9%) | 52/323 (16.1%) | **RR 0.78** (0.51 to 1.20) | **35 fewer per 1.000** (from 79 fewer to 32 more) | ⨁⨁⨁⨁ High |  |

**CI:** confidence interval; **RR:** risk ratio
